# Viscoelastic and Functional Properties of Cod-Bone Gelatin in the Presence of Xylitol and Stevioside

**DOI:** 10.3389/fchem.2018.00111

**Published:** 2018-05-22

**Authors:** Linyu Nian, Ailing Cao, Jing Wang, Hongyu Tian, Yongguo Liu, Lingxiao Gong, Luyun Cai, Yanbo Wang

**Affiliations:** ^1^College of Food Science and Engineering, Bohai University, National & Local Joint Engineering Research Center of Storage, Processing and Safety Control Technology for Fresh Agricultural and Aquatic Products, Jinzhou, China; ^2^Beijing Advanced Innovation Center for Food Nutrition and Human Health, Beijing Technology and Business University, Beijing, China; ^3^Hangzhou Customs District, Hangzhou, China; ^4^College of Food Science and Biotechnology, Zhejiang Gongshang University, Hangzhou, China

**Keywords:** bone gelatin, xylitol, stevioside, *Gadus morhua*, cod

## Abstract

The physical, rheological, structural and functional properties of cod bone gelatin (CBG) with various concentrations (0, 2, 4, 6, 10, and 15%) of low-calorie sweeteners [xylitol (X) and stevioside (S)] to form gels were investigated. The gel strength of CBGX increased with increased xylitol due presumably to hydrogen bonds between xylitol and gelatin, but with CBGS the highest gel strength occurred when S concentration was 4%. Viscosity of CBGS samples were higher than CBGX due to S's high molecular mass. The viscoelasticity (G′ and G′′), foaming capacity and fat binding capacity of CBGX were higher while foam stability was lower. The emulsion activity and emulsion stability of CBGX were a little lower than CBGS at the same concentration. The structure of X is linear making it easier to form a dense three-dimensional network structure, while the complex cyclic structure of S had more difficulty forming a network structure with cod bone gelatin. Therefore, X may be a better choice for sweetening gelatin gels.

## Introduction

Gelatin is widely used in foods, pharmaceuticals, and cosmetics because of its functional properties, such as elasticity, stability, emulsibility, foamability, thickening, and gelling properties (Chandra et al., 2013; Alfaro et al., 2015). Skins and bones from pigs and cows have usually been used for confectionery and jelly products (Sai-Ut et al., 2012), but bovine spongiform encephalopathy (BSE) and foot and mouth disease (FMD) along with religious concerns of Muslims, Jews, Hindus, and Buddhists has led to more interest in using aquatic by-products such as fish skins, bones, scales, heads, and connective tissues as a potential source of gelatin. Bones and skins together are about ~30% of the whole fish (Peinado et al., 2016).

Cod (*Gadus morhua*) species, e.g., *Gadus morhua, Gadus ogac*, and *Gadus macrocephalus* are among the most important commercial species and are caught in both the Atlantic and Pacific oceans (Thorarinsdottir et al., 2010). Tons of cod bones are discarded or, at best, processed into feeds. In this paper cod bone gelatin will be further studied.

Low-calorie sweeteners including natural sweeteners such as stevioside and xylitol provide a sweet taste with no or few calories (Kroger et al., 2006). Stevioside is obtained from the leaves of *Stevia rebaudiana* plants. Its relative sweetness is about 200–300 times that of sucrose with no acute or subacute toxicity, genotoxicity or carcinogenicity (Lemusmondaca et al., 2012). As a functional sweetener, xylitol is applied to some low-sugar or sugar-free foods. Its sweetness is about the same as sucrose, and it can inhibit some bacteria from growing, prevent the formation of plaque, reduce the acid-producing ability of bacteria and decrease the adsorption of bacteria on the surface of teeth. Thus xylitol helps prevent dental caries (Islam, 2011). These two sweeteners are not digested so have zero calories, which is helpful for diabetic patients (Jo and Surh, 2015).

The application of low calorie sweeteners in foods is increasingly important as they are used as a substitute for sucrose (Sharma et al., 2016). In the present paper, the physical, rheological, structural and functional properties of cod bone gelatin with xylitol and stevioside was studied.

## Materials and methods

### Materials

Cod (*Gadus morhua*) back bones were directly obtained from Tianbao Green Food Co., Ltd. (Dalian, Liaoning, China), and the residual meat and connective tissues of bones were removed using a knife. The fish bones (around 2 cm length) were kept at 4°C for a maximum of 1 day until the bone gelatin extraction. Xylitol was obtained from Shandong Longli Bio Technologies Inc. (Qingdao, Shandong, China). Stevioside was provided by Niutang Chemical Co., Ltd. (Changzhou, Jiangsu, China). All other chemicals used were obtained from Sinopharm Chemical Reagent Co., Ltd., and at least of analytical grade.

### Methods

#### Gelatin extraction of cod bones

The back bones were rinsed with distilled water, and the gelatin extraction was done as described by Mahmoodani et al. (2014) with slight modifications. Briefly, the bones were soaked in 0.1 mol/L NaOH at a ratio of 1:15 (w/v) to remove pigments and non-collagenous proteins, with magnetic stirring at room temperature (25°C) for 4 H. The above solution was changed every h. After that, the samples were washed with distilled water until a neutral pH (7.0) using a pH meter (PHS-3C, Shanghai Yidian Technology Instrument Co., Ltd., Shanghai, China) and the liquid was removed by cotton gauze. After the alkaline treatment, the bones were mixed with 0.2 mol/L EDTA for 12 h to decalcify. Then the bones were mixed with 0.1 mol/L HCl at a ratio of 1:15 (w/v) and stirred at room temperature for 1 h. The acid treatment can destroy ionic bonds between collagen molecules and increase the extraction rate (Hattrem et al., 2015). The bones were washed with distilled water to neutral pH. Subsequent gelatin extraction was done in distilled water at 55–70°C for 4 h, increasing 5°C every h. The extracted solution was centrifuged at 10,000 × g (Sorvall Stratos Centrifuge, Thermo Fisher Scientific, Waltham, MA, USA) for 30 min at 4°C and the supernatant was dried in a freeze-dryer (LG-1.0, Xinyang Quick-freezing Equipment Manufacturing Co., Ltd., Shenyang, China) for 72 h.

#### Sample preparations

Cod bone gelatin powder (6.67 g) was dissolved in distilled water to obtain 100 mL of solution. About 0, 2, 4, 6, 10, 15% (w/v) of xylitol or stevioside powder, respectively, was added and the final concentration of gelatin in the mixture was kept to a constant value of 6.67%. Samples were heated at 35°C for 30 min in a water bath (HH-6, Changzhou Guohua Electric Appliance Co., Ltd., Jiangsu, China), and stirred until well blended. The mixtures (20 mL) were poured into plastic cups (30 mm diameter × 20 mm height, flat bottomed). They were cooled to room temperature, and then refrigerated at 4°C for 18 h for hydrogel formation. These were used to measure physical, rheological and functional properties. The xerogels after freeze-drying were used to determine microstructure.

#### Determination of gelatin yield

Gelatin yield on the dry and wet basis were calculated according to the method of Du et al. (2013):

$$\begin{array}{l} \text{Gelatin yield }\left(\text{\%}\right)=100\times \frac{\text{Dry weight of extracted gelatin }\left(\text{g}\right)}{\text{Dry weight of cod bones }\left(\text{g}\right)} \end{array}$$

$$\begin{array}{l} \text{Gelatin yield }\left(\text{\%}\right)=100\times \frac{\text{Dry weight of extracted gelatin }\left(\text{g}\right)}{\text{Wet weight of cod bones }\left(\text{g}\right)} \end{array}$$

In actuality this is a total powder yield assuming that the powder is protein only and in particular gelatin.

### Determination of physical properties

#### Gel strength

Gel strength was determined using a TA-XT Plus texture profile analyser (Stable Micro Systems Ltd., Godalming, UK) using the method of Cai et al. (2016). A P/0.5 plastic probe with 12.7 mm diameter was used with a 5.0 g trigger force. The gel strength of all gel products were tested until the samples center were pressed into 4 mm at the speed of 1 mm/s, and the maximum stress was the value of gel strength. The experiments were done at least in triplicate.

#### Viscosity

Viscosity of gelatin solutions was measured using a rheometer (Discovery HR-1, TA Instrument Ltd., New Castle, DE, USA) equipped with a cone plate geometry (Guo et al., 2015). The diameter was 40 mm, the cone angle was 2°, and the truncation gap was 5 mm. A 1 mL gelatin solution at 35°C was applied to the plate and the excess sample was removed. The sample was left for 20 s, and then the gelatin solution's shear rate was linearly increased from 0.1 to 100 s^−1^ in 2 min. The shear viscosity was expressed as Pa.s obtained from the computer program with the instrument.

### Determination of rheological properties

#### Temperature sweeps

The dynamic rheological properties of the gels using the method of Anvari and Chung (2016) with slight modifications was done using the rheometer. The samples were at 35°C for 30 min using the same geometry as previously with a strain of 2% and a frequency of 1 Hz. The dynamic rheological properties were measured from 25 to 0°C with a cooling rate of 1°C/min. After cooling, gelatins were kept at 0°C for 1 min and then heated back to 25°C at the same rate. During the measurement, the sample was covered with paraffin to prevent evaporation. The G′ (elastic or storage modulus) and G′′ (viscous or loss modulus) values as a function of temperature gave the viscoelastic properties of cod bone gelatin with and without the low-calorie sweeteners.

#### Frequency sweeps

The dynamic frequency sweep test was done using the method of Huang et al. (2017). After the temperature sweep samples were cooled to 4°C, strain was held at 2%, and the frequency ranged from 0.1 to 10 Hz. The G′ and G′′ were determined as a function of frequency.

#### Determination of structural properties

Scanning electron microscope (SEM) was used to observe the microstructure of gelatin mixtures. The gelled samples (0.5 × 0.5 × 0.5 cm^3^) were dissolved in glutaraldehyde solution (2.5%, v/v) to fix at 4°C for 24 h, then rinsed using 0.2 mol/L sodium phosphate buffer solution (pH 6.8) three times for 10 min each. After that a graduate ethanol solution was used at 30, 50, 60, 70, 80, 90, and 100% for 10 min each, then samples were dried in the freeze-dryer for 36 h, gold coated (E-1045 Ion sputter, Hitachi Ltd., Tokyo, Japan) and viewed in the SEM (S-4800, Hitachi Ltd., Tokyo, Japan) at an acceleration voltage of 3 kV and 5,000× magnification to observe the surface structure.

### Determination of functional properties

#### Foaming properties

The foaming properties including the foam capacity (FC) and foam stability (FS) were determined using the method of Kittiphattanabawon et al. (2012) with sight modifications. The CBGX and CBGS solution (1%; 100 mL) with different concentrations of sweeteners were transferred into a 250 mL cylinder (70 mm in diameter) and the mixtures were homogenized for 1 min (FJ 300-SH, Shanghai Model Sample Co., Ltd., Shanghai, China) at 4,000 rpm at room temperature (25°C). The height of the foam and liquid surface were measure at times 0, 30, and 60 min. FC and FS were calculated using the following equations:

$$\begin{array}{lll} \text{FC }\left(\text{\%}\right) & = & \frac{{\text{V}}_{\text{T}}}{{\text{V}}_{0}}\times 100\\ \text{FS }\left(\text{\%}\right) & = & \frac{{\text{V}}_{\text{t}}-{\text{V}}_{0}}{{\text{V}}_{0}}\times 100 \end{array}$$

Where V_T_ refers to the total volume after homogenizing (mL), V_0_ to the initial volume of solution, V_t_ to the both liquid and foam heights after leaving at room temperature for 30 and 60 min.

#### Emulsifying properties

Emulsion activity index (EAI) and emulsion stability index (ESI) were done as described by Kittiphattanabawon et al. (2012) and Waniska and Kinsella (1988) with a slight modification. Gelatin was dissolved in sweetener solution with different concentrations to obtain a final concentration of 1% (w/v). To prepare the emulsion, soybean oil (2 mL) was added to 6 mL of the gelatin solution and then the mixtures were homogenized at 6,000 rpm for 1 min at room temperature.

The emulsion was diluted with 0.1% sodium dodecyl sulfate (SDS) to obtain a dilution factor of 100, and the absorbance was measured immediately (A_0_) and 10 min (A_10_) after emulsification at 500 nm using a spectrophotometer (UV-2550, Shimadzu Suzhou Instruments Mfg. Co., Ltd., Kyoto, Japan). EAI and ESI were calculated using the following equations:

$$\begin{array}{l} \text{EAI }\left({\text{m}}^{2}/\text{g}\right)=2\times \text{T}\times \frac{\text{A}0\times \text{DF}}{\text{Cl }\Phi }\\ \text{ESI }\left(\text{min}\right)=\frac{\text{A}0\times \text{t}}{\text{A}0-\text{A}10} \end{array}$$

Where t = 10 min, T = 2.303, DF = dilution factor (100), C = protein concentration in the aqueous phase (10 g/m^3^), l = path length of cuvette in meters (0.01 m), Φ = oil phase volume fraction (0.25).

#### Water holding capacity and fat binding capacity

Water holding capacity (WHC) and fat binding capacity (FBC) were determined using the method of Nurul and Sarbon (2015) with slight modifications. For WHC, 0.5 g gelatin was dissolved in 10 mL distilled water, and then added with different concentrations low-calorie sweeteners, and the mixed solution were stirred with glass rod for 30 s every 15 min till to 1 h. The samples were centrifuged at 3,000 × g for 25 min at room temperature to obtain the supernatant. The supernatant was filtered through Whatman grade No. 1 filter paper (Shanghai Mosu Science Equipment Co., Ltd., Shanghai, China) after tilting to a 45° angle, and the volume was measured accurately. FBC was done using 10 ml of palm oil and mixed for only 10 min. Otherwise the steps were the same. The WHC and FBC were calculated using the following equations:

$$\begin{array}{l} \text{WHC},\text{ FBC }\left(\text{mL}/\text{g}\right)=\frac{{\text{V}}_{0}-{\text{V}}_{1}}{\text{Weight of gelatin}} \end{array}$$

Where V_0_ refers to initial volume, V_1_ to the volume of the supernatant and the Weight of gelatin to the total powder.

#### Statistical analysis

All data were obtained in triplicate to use to calculate mean values and standard deviations, which were compared using one-way analysis of variance (ANOVA). Duncan's multiple range test was used to show the differences among mean values at a significance level of 0.05 using SPSS 19 for Windows (SPSS Inc., Chicago, IL, USA). Origin Pro 8.5 (OriginLab, USA) was used for diagramming.

## Results and discussion

### The yield of cod bone gelatin

The yield of cod bones gelatin was 7.9 g and 2.0 g/100 g on the basis of dry and wet weight of the bones, respectively.

### Analysis of physical properties

#### Gel strength

The gel strength of gelatin gels with various concentrations of xylitol and stevioside are shown in Table 1. The gel strength of CBGX was significantly (*P* < 0.05) increased as xylitol concentration increased indicating a positive interaction between the gelatin and the xylitol. The stevioside and gelatin had the highest gel strength at 4% stevioside was 4% suggesting an initial positive interaction followed by a negative interaction similar to that observed by Wangtueai et al. (2010).

**Table 1:** Gel strength and functional properties [foam capacity (FC), foam stability (FS), emulsion activity index (EAI), emulsion stability index (ESI), water holding capacity (WHC) and fat binding capacity (FBC)] of cod bone gelatin with different concentrations of xylitol and stevioside^a^^,^^b^.

**Concentration**	**Gel strength (g)**		**FC (%)**		**FS (%)**			
					**30 min**		**60 min**	
	**FGBX**	**FGBS**	**FGBX**	**FGBS**	**FGBX**	**FGBS**	**FGBX**	**FGBS**
0%	185.3 ± 6.16^f^	185.3 ± 6.16^c^	189.2 ± 0.46^a^	189.2 ± 0.15^a^	89.8 ± 1.00^e^	89.8 ± 0.28^f^	84.6 ± 0.32^e^	84.6 ± 0.27^f^
2%	200.3 ± 8.54^e^	192.9 ± 6.88^b^	185.6 ± 0.26^b^	181.3 ± 0.17^b^	92.3 ± 0.64^d^	92.5 ± 0.01^e^	88.4 ± 0.25^d^	88.9 ± 0.01^e^
4%	216.8 ± 8.58^d^	211.0 ± 2.81^a^	179.2 ± 0.06^c^	174.5 ± 0.61^c^	92.9 ± 0.19^d^	93.4 ± 0.07^d^	88.9 ± 0.35^d^	89.7 ± 0.00^d^
6%	223.5 ± 10.75^c^	183.6 ± 3.56^c^	170.0 ± 0.04^d^	163.9 ± 0.20^d^	94.0 ± 0.00^c^	94.9 ± 0.06^c^	90.1 ± 0.02^c^	90.4 ± 0.03^c^
10%	245.8 ± 4.96^b^	167.6 ± 6.28^d^	160.2 ± 0.85^e^	154.4 ± 0.12^e^	96.3 ± 0.08^b^	96.8 ± 0.04^b^	92.0 ± 0.15^b^	92.6 ± 0.1^b^
15%	254.8 ± 6.90^a^	140.3 ± 5.76^e^	139.3 ± 0.03^f^	126.3 ± 0.00^f^	98.2 ± 0.13^a^	98.9 ± 0.01^a^	93.1 ± 0.08^a^	93.9 ± 0.02^a^
**Concentration**	**EAI (m**^2^**/g)**		**ESI (min)**		**WHC (mL/g)**		**FBC (mL/g)**	
	**FGBX**	**FGBS**	**FGBX**	**FGBS**	**FGBX**	**FGBS**	**FGBX**	**FGBS**
0%	20.43 ± 0.61^e^	20.43 ± 0.94^e^	22.15 ± 0.21^e^	22.15 ± 0.14^e^	4.01 ± 0.01^f^	4.01 ± 0.04^c^	8.89 ± 0.02^a^	8.89 ± 0.05^a^
2%	23.89 ± 0.38^c^	23.24 ± 0.86^c^	25.67 ± 0.08^c^	25.28 ± 0.06^c^	6.07 ± 0.02^e^	5.92 ± 0.01^b^	7.90 ± 0.37^b^	7.04 ± 0.67^b^
4%	25.76 ± 1.00^b^	25.17 ± 0.92^b^	27.59 ± 0.10^b^	27.16 ± 0.02^b^	7.92 ± 0.16^d^	5.99 ± 0.05^b^	7.83 ± 0.52^bc^	6.89 ± 0.82^b^
6%	28.12 ± 0.00^a^	28.06 ± 1.00^a^	29.24 ± 0.00^a^	28.93 ± 0.05^a^	9.35 ± 0.10^c^	6.03 ± 0.10^b^	7.51 ± 0.22^bcd^	6.73 ± 0.56^b^
10%	22.52 ± 0.40^d^	22.27 ± 0.01^d^	23.97 ± 0.40^d^	23.22 ± 0.01^d^	11.04 ± 0.12^b^	7.09 ± 0.02^a^	7.30 ± 0.19^cd^	6.35 ± 0.49^b^
15%	17.20 ± 0.82^f^	16.96 ± 0.86^f^	18.15 ± 0.12^f^	18.02 ± 0.16^f^	12.82 ± 0.03^a^	7.11 ± 0.35^a^	7.02 ± 0.28^d^	6.27 ± 0.11^b^

a *All values were means ± standard deviation of three values*.

b *Different small letters in the same column indicate significant differences between means (P < 0.05)*.

#### Viscosity

**Figure 1:**
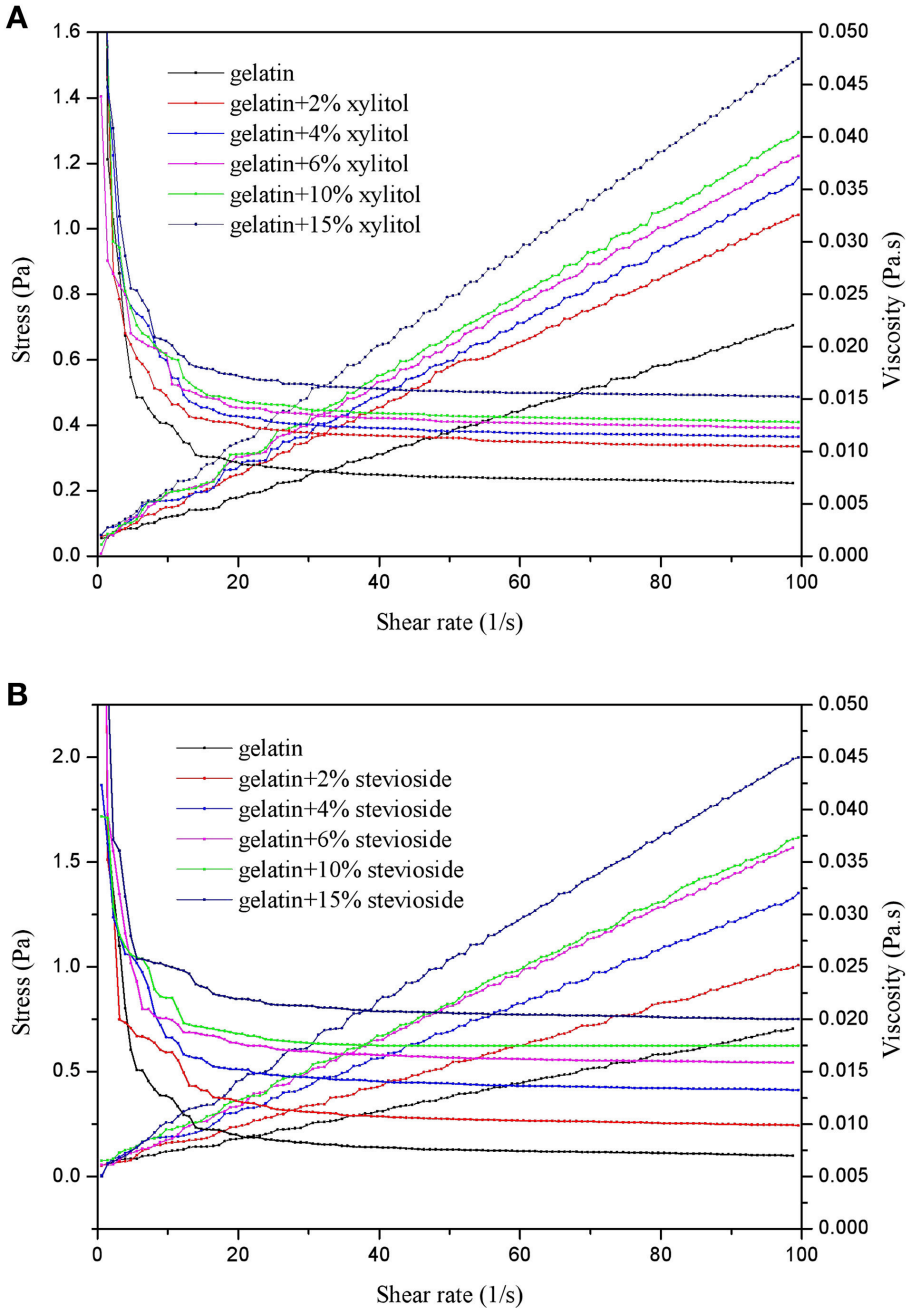
Shear stress and viscosity of cod bone gelatin with addition of xylitol **(A)** and stevioside **(B)**.

The effects of xylitol and stevioside on the shear stress and viscosity of cod bone gelatin is shown in Figure 1. As shown in Figures 1A,B, the shear stress of CBGX and CBGS were linearly dependent on the shear rate. The viscosity was unchanged at the high shear rates (10–100 s^−1^) when xylitol and stevioside were added to the gelatin. The samples showed Newtonian behavior (Yang and Wang, 2009). The viscosity of CBGX and CBGS decreased rapidly at shear rates of 0–10 s^−1^ and non-Newtonian (Bingham) fluid was observed at low shear rate. The shear stress and viscosity of gelatin solution gradually increased as the concentration of xylitol and stevioside increased. The gel solutions thickened and the shear resistance increased with increased xylitol and stevioside. The viscosity of CBGS was slightly higher than CBGX. The molecule mass of stevioside is 805 Da which is higher than xylitol at 152 Da which may help account for the higher viscosity of CBGS.

### Analyses of dynamic rheological properties

#### Temperature sweeps

**Figure 2:**
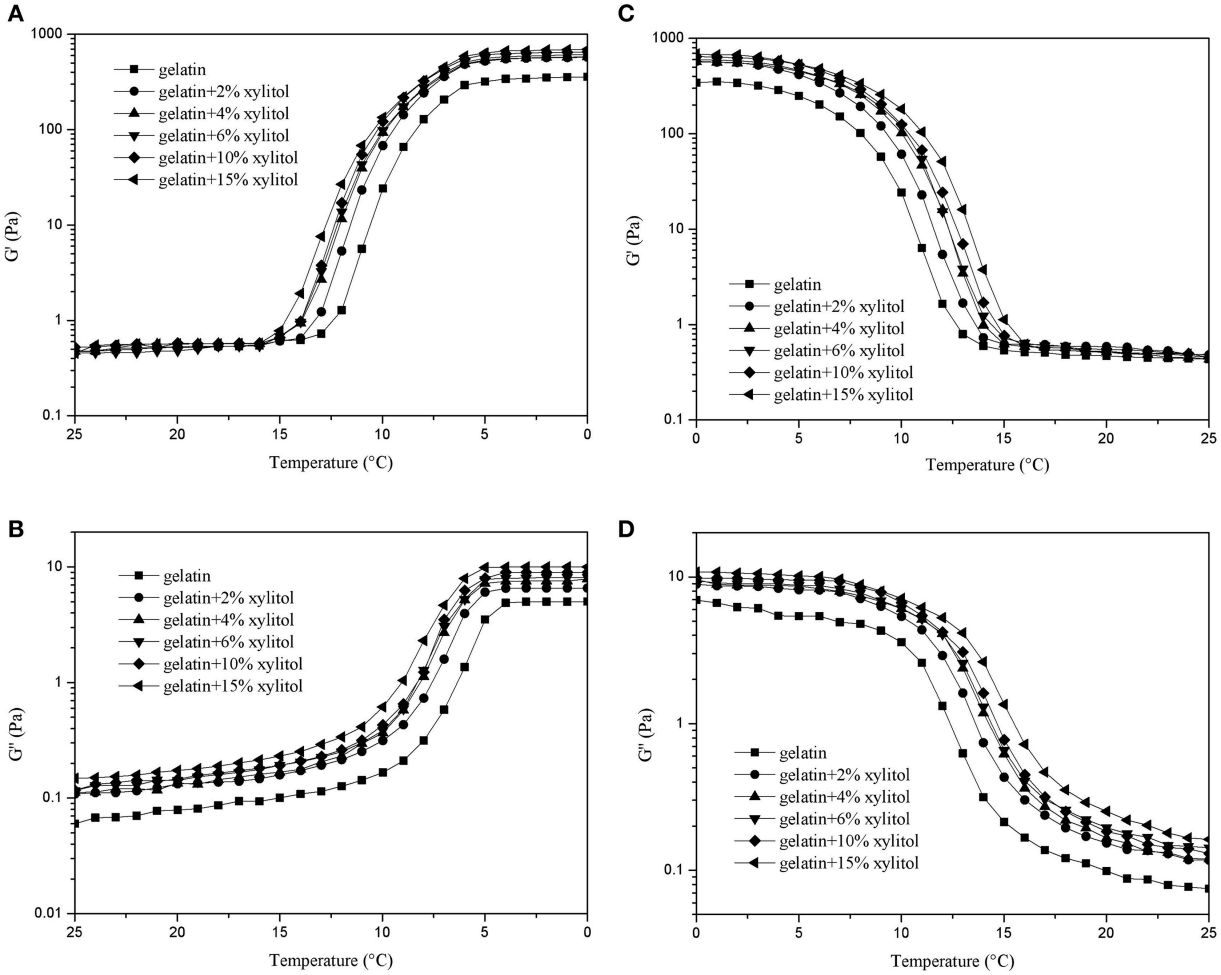
G′ and G″ of cod bone gelatin with various concentrations of xylitol of during cooling **(A,B)** and heating **(C,D)**.

Gelatin's elastic properties allow it to store energy in the gel state, and its viscous properties allows it to dissipated energy in the solution state (Tau and Gunasekaran, 2016). Figure 2 shows the dynamic rheological properties of CBGX during cooling (Figures 2A,B) and heating (Figures 2C,D). During cooling, G′ and G′′ of the gelatin gels increased rapidly between 12 and 6°C, which represented the transition from solution to gel state. During heating, a rapid decrease of G′ and G′′ was observed from 7 to 13°C, which represented the transition from gel to solution state.

As shown in Figure 2, the values of G′ for the mixed systems were higher than G′′, which indicates that the elastic behavior of the system was greater than the viscous behavior. CBG and CBGX had a low slope of curve at low temperature, which indicated a good gelling ability (Goudoulas and Germann, 2017). As xylitol concentration increased, the G′ and G′′ of CBGX increased gradually and was higher than CBG with both cooling and heating indicating that xylitol stabilized the molecular structure of the cod bone gelatin.

**Figure 3:**
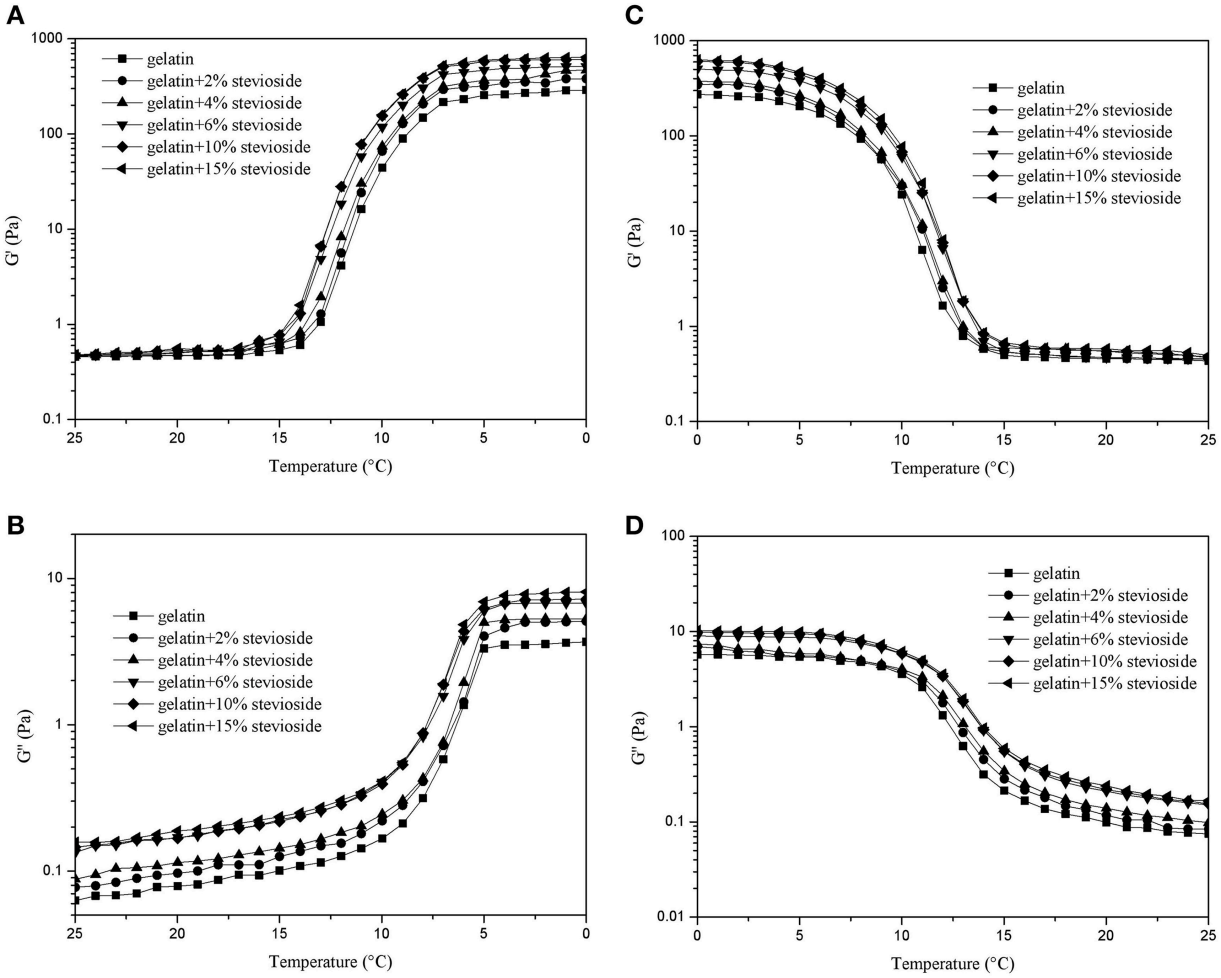
G′ and G″ of cod bone gelatin with various concentrations of stevioside during cooling **(A,B)** and heating **(C,D)**.

Figure 3 shows the dynamic rheological properties of CBGS during cooling (Figures 3A,B) and heating (Figures 3C,D). The G′ values were 10 times higher than G′′. With increased concentration of stevioside, G′ and G′′ increased gradually and were higher than those of CBG. The gelling temperature varied from 10 to 5°C for CBGS, which was a little lower than CBGX, while the melting temperature was similar to CBGX and ranged from 7 to 13°C. The effect of stevioside on the dynamic rheological properties of cod bone gelatin were similar to xylitol, with both improving the stability of the gelatin system. Stevioside and xylitol have the same number of hydroxyl groups that are available to form hydrogen bonds with the cod bone gelatin carboxyls (Cai et al., 2017), which would stabilize the molecular structure of fish gelatin and increase the system's viscous resistance and elastic properties.

#### Frequency sweeps

**Figure 4:**
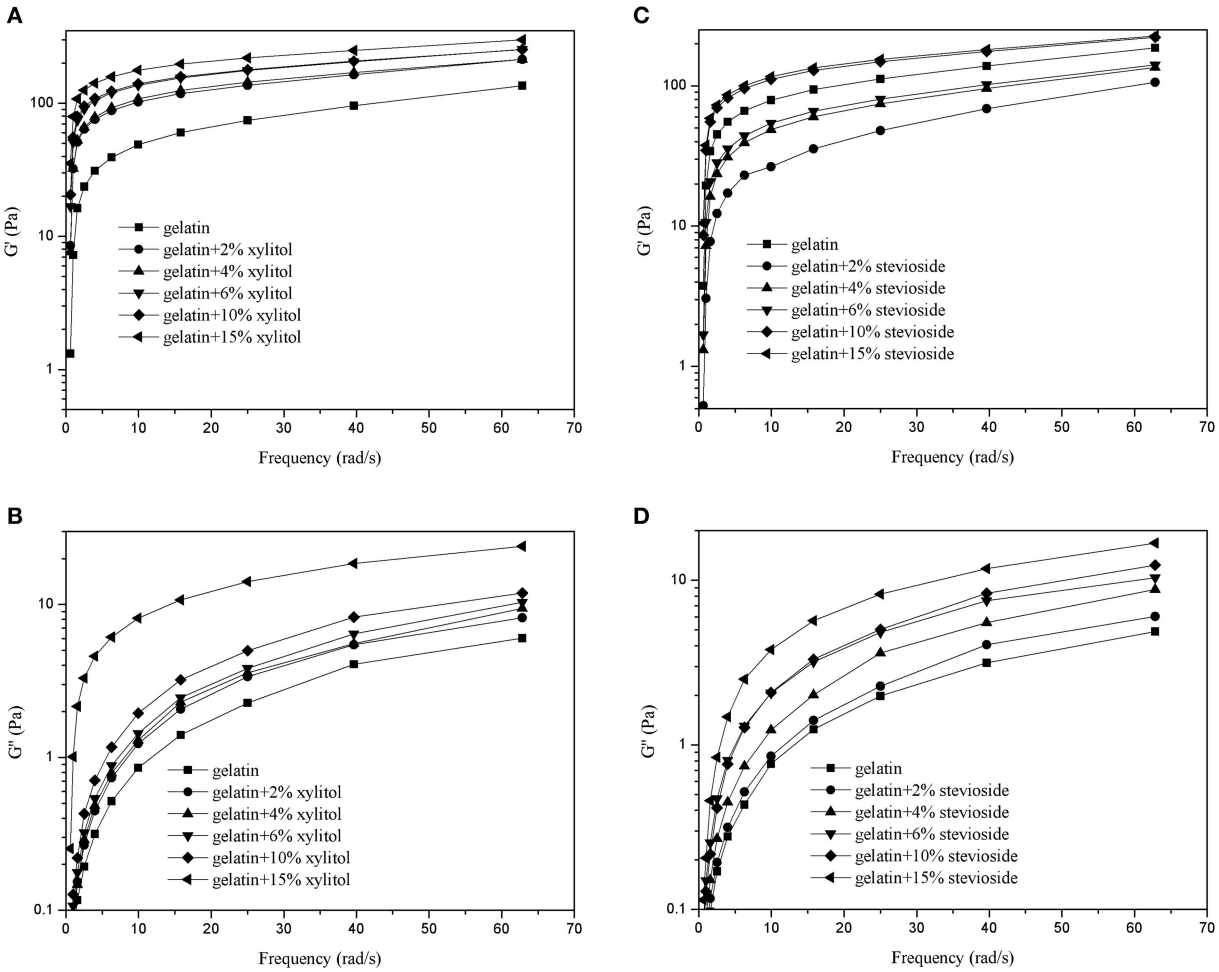
G′ and G″ of cod bone gelatin with various concentrations of xylitol **(A,B)** and stevioside **(C,D)** during the frequency sweeps.

Figure 4 shows the frequency sweep results for the G′ of CBGX (Figure 4A) and CBGS (Figure 4C), and G′′ of CBGX (Figure 4B) and CBGS (Figure 4D). As shown in Figure 4, the values of G′ were higher than G′′ due to the formation of the elastic hydrogel described above. The G′ and G′′ of CBGX and CBGS increased with increased xylitol or stevioside concentration, which was consistent with the temperature sweeps.

#### Analyses of microstructure

**Figure 5:**
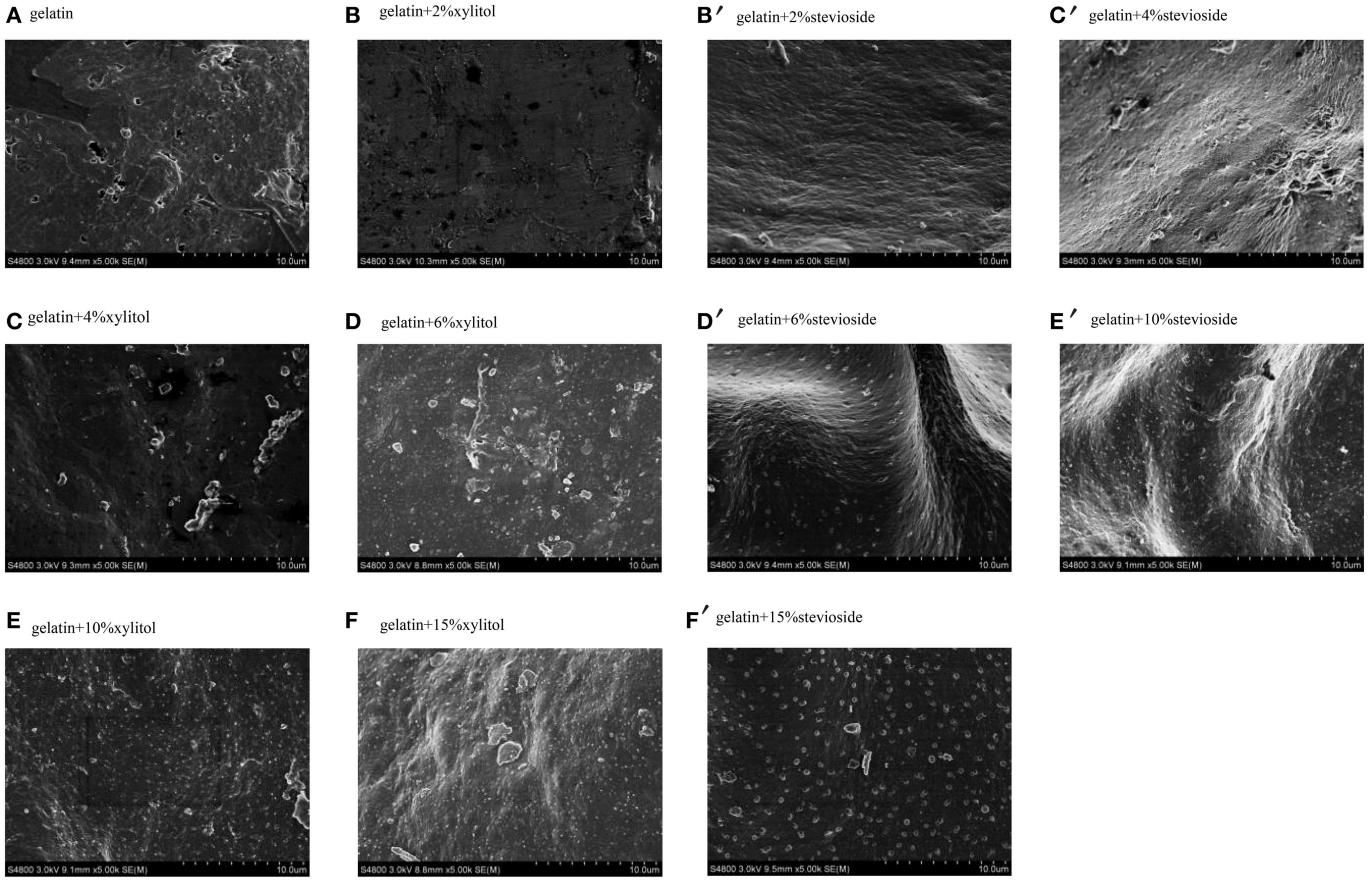
Microstructure of cod bone gelatin with various concentrations of xylitol and stevioside.

Figure 5 shows the SEM micrographs which reflects the surface morphology of the gels. The cod bone gelatin had a much looser network, larger voids, irregular gatherings and many small holes between the protein molecules compared with the gels with xylitol and stevioside. With xylitol or stevioside, the three-dimensional network structure became more compact and ordered, and as concentration increased, the number and size of voids were significantly reduced. The CBGX tended to form a homogeneous three-dimensional network structure which was cross-linked by linear chains. However, there were more intense and stronger interactions between cod bone gelatin and stevioside molecules which led to a rougher microstructure and coarse crosslinked chains. With higher concentration, the structures became denser with more sweetener molecules on the surface of the gelatin.

### Analyses of functional properties

#### Foaming properties

FC and FS are shown in Table 1. The FC of CBGX and CBGS decreased significantly (*P* < 0.05) with increasing concentration. This was probably due to the increased viscosity of the whole system making it more difficult to develop the interface needed to create a foam (Sila et al., 2015). The FS of CBGX and CBGS increased significantly (*P* < 0.05), but there were no significant differences (*P* > 0.05) at 2 and 4% xylitol after standing for 30 or 60 min. The FC values of CBGX were higher than CBGS while the values of FS were lower, possibly because the stevioside may have had a reduce surface tension.

#### Emulsifying properties

The EAI and ESI are shown in Table 1. EAI and ESI of CBGX and CBGS had similar trends and increased significantly (*P* < 0.05) up to 6% concentration as also reported by Sila et al. (2015). The values of CBGX and CBGS were lower than CGB when the concentration of sweeteners was 15%. The sweeteners changed the flowability because of the increased viscosity which has been shown to increase emulsion activity and emulsion stability (Aewsiri et al., 2013). The EAI and ESI of CBGX were a little lower than CBGS at the same concentration. This may be partially attributed to molecular weight and the additional stability with stevioside.

#### Water holding and fat binding capacities

Water holding and fat binding capacities (Table 1) are interfacial properties that reflect the interactions between water, oil and gelatin and sweetner (Jeya et al., 2012). The WHC increased significantly (*P* < 0.05) after the addition of xylitol or stevioside. The increase in the gel structure might increase the WHC and the large number of hydroxyl groups also could increase the WHC. However, the change with concentration of CBGX was steep while the trend for CBGS was less steep so that the WHC of CBGX were higher than CBGS at the same concentration.

The FBC increased significantly (*P* < 0.05) when the concentration of xylitol went from 2 to 4%, and from 10 to 15%. The FBC of CBGS was not significantly (*P* > 0.05) increased with stevioside concentrations from 0 to10%, but was significant higher at 15%. The values of CBGX were higher than CBGS at the same concentration, which was similar to WHC.

## Conclusion

The linear structure of xylitol seemed to make it easier to form a stronger, denser three-dimensional network structure, while the complex, cyclic structure of stevioside made it more difficult to form a network structure with cod bone gelatin. Therefore, xylitol has a greater potential to be used with gelatin food products to both provide a good structure and non-caloric sweetness.

## Author contributions

LC and LN contributed conception and design of the study. LN and AC organized the database. LN, RG, JL, and YZ performed the statistical analysis and wrote the first draft of the manuscript. LC, LN, and JX wrote sections of the manuscript. All authors contributed to manuscript revision, read and approved the submitted version.

### Conflict of interest statement

The authors declare that the research was conducted in the absence of any commercial or financial relationships that could be construed as a potential conflict of interest.
