# Corrigendum: Viscoelastic and Functional Properties of Cod-Bone Gelatin in the Presence of Xylitol and Stevioside

**DOI:** 10.3389/fchem.2019.00120

**Published:** 2019-04-02

**Authors:** Linyu Nian, Ailing Cao, Jing Wang, Hongyu Tian, Yongguo Liu, Lingxiao Gong, Luyun Cai, Yanbo Wang

**Affiliations:** ^1^College of Food Science and Engineering, Bohai University, National & Local Joint Engineering Research Center of Storage, Processing and Safety Control Technology for Fresh Agricultural and Aquatic Products, Jinzhou, China; ^2^Beijing Advanced Innovation Center for Food Nutrition and Human Health, Beijing Technology and Business University, Beijing, China; ^3^Hangzhou Customs District, Hangzhou, China; ^4^College of Food Science and Biotechnology, Zhejiang Gongshang University, Hangzhou, China

**Keywords:** bone gelatin, xylitol, stevioside, *Gadus morhua*, cod

In the published article, an author name was incorrectly spelled as “Yuhao Wang.” The correct spelling is “Yanbo Wang.”

Additionally, there was an error in affiliation 2. Instead of “Xiaoshan Entry-Exit Inspection and Quarantine Bureau, Hangzhou, China,” it should be “Hangzhou Customs District, Hangzhou, China.”

Lastly, there was also an error regarding the affiliations for Linyu Nian and Luyun Cai. They should also have the affiliation “Beijing Advanced Innovation Center for Food Nutrition and Human Health, Beijing Technology and Business University, Beijing, China” and Yanbo Wang should not have affiliation 3.

The authors apologize for this error and state that this does not change the scientific conclusions of the article in any way. The original article has been updated.

